# More deterministic assembly constrains the diversity of gut microbiota in freshwater snails

**DOI:** 10.3389/fmicb.2024.1394463

**Published:** 2024-07-08

**Authors:** Zhaoji Shi, Fucheng Yao, Qi Chen, Yingtong Chen, Jiaen Zhang, Jing Guo, Shaobin Zhang, Chunxia Zhang

**Affiliations:** ^1^College of Natural Resources and Environment, South China Agricultural University, Guangzhou, China; ^2^Guangdong Engineering Technology Research Centre of Modern Eco-Agriculture and Circular Agriculture, South China Agricultural University, Guangzhou, China; ^3^Guangdong Provincial Key Laboratory of Eco-Circular Agriculture, South China Agricultural University, Guangzhou, China; ^4^Key Laboratory of Agro-Environment in the Tropics, Ministry of Agriculture and Rural Affairs, South China Agricultural University, Guangzhou, China; ^5^Henry Fok School of Biology and Agriculture, Shaoguan University, Shaoguan, China

**Keywords:** community assembly, biological invasion, *Pomacea canaliculata*, Viviparidae, freshwater snail

## Abstract

Growing evidence has suggested a strong link between gut microbiota and host fitness, yet our understanding of the assembly mechanisms governing gut microbiota remains limited. Here, we collected invasive and native freshwater snails coexisting at four independent sites in Guangdong, China. We used high-throughput sequencing to study the assembly processes of their gut microbiota. Our results revealed significant differences in the diversity and composition of gut microbiota between invasive and native snails. Specifically, the gut microbiota of invasive snails exhibited lower alpha diversity and fewer enriched bacteria, with a significant phylogenetic signal identified in the microbes that were enriched or depleted. Both the phylogenetic normalized stochasticity ratio (pNST) and the phylogenetic-bin-based null model analysis (iCAMP) showed that the assembly process of gut microbiota in invasive snails was more deterministic compared with that in native snails, primarily driven by homogeneous selection. The linear mixed-effects model revealed a significant negative correlation between deterministic processes (homogeneous selection) and alpha diversity of snail gut microbiota, especially where phylogenetic diversity explained the most variance. This indicates that homogeneous selection acts as a filter by the host for specific microbial lineages, constraining the diversity of gut microbiota in invasive freshwater snails. Overall, our study suggests that deterministic assembly-mediated lineage filtering is a potential mechanism for maintaining the diversity of gut microbiota in freshwater snails.

## 1 Introduction

The animal gut houses a wide range of microbes (Bankers et al., 2021; Levin et al., 2021), which play crucial roles in various aspects of the host digestion (Francoeur et al., 2020), detoxification (Levin et al., 2021), and immune system (Xiong et al., 2019). Despite the importance of these functions for most animals (Moran et al., 2019), the processes that shape gut microbiota remain largely unexplored, particularly in the case of freshwater snails. Understanding the mechanisms shaping snail gut microbiota is a crucial first step in developing microbial approaches to control invasive snails and preserve native snails.

After years of research, we have gained some insights into the gut microbiota of freshwater snails, with a primary focus on their diversity, composition, and functions (Liu et al., 2018; Chen et al., 2021). Studies have revealed that the gut microbiota in freshwater snails is shaped by both host-related factors, such as species (Zhou et al., 2022), sexes (Chen et al., 2021), and developmental stages (Chen et al., 2021), and environmental factors like season (Li et al., 2022b), temperature (Li et al., 2022a), and toxins (Liu et al., 2023). These studies have systematically uncovered the variations in the gut microbiota of freshwater snails. Among these, the invasive apple snail *Pomacea canaliculata* attracts attention not only as a pest of rice (Halwart, 1994; Attademo et al., 2016) but also for its ecological advantages in invaded habitats (Fang et al., 2016; Liu et al., 2021b). Research on this snail indicates that its gut microbiota may facilitate adaptation to new environments (Li et al., 2022b; Zhou et al., 2022), thereby providing a basis for developing invasive management strategies that leverage snail gut microbiota. However, there is still much to be explored to harness the potential of gut microbiota, such as understanding the assembly processes of freshwater snail gut microbiota.

According to the current consensus of community assembly, both deterministic and stochastic processes jointly govern the assembly of communities (Zhou and Ning, 2017; Ning et al., 2019, 2020). The neutral theory highlights the stochastic processes, like random birth or death events, immigration, and ecological drift (Chave, 2004), while the niche-based theory assumes that deterministic processes, including environmental filtering and biological interactions (Chesson, 2000). Quantifying the importance of these two processes in governing microbiota assembly has sparked significant scientific interest (Liu et al., 2020; Wang et al., 2020; Ge et al., 2021). In the study of gut microbiota, these processes have also been extensively investigated (Burns et al., 2016; Zhu et al., 2016; Xu et al., 2023) and are considered ecology and evolution significant (Kohl, 2020; Ge et al., 2021). For instance, researchers have found that changes in the importance of these processes can effectively explain the differences in the composition of gut microbiota from different honeybee species and geographical sites (Ge et al., 2021). However, there has been limited research focusing on the potential association between community assembly and gut microbiota diversity. The diversity of animal gut microbiota is thought to be influenced by host filtering, which includes factors such as the immune system and stomach pH (Reese and Dunn, 2018). Host filtering functions as the host's selection for specific microbes (Ley et al., 2006; Kohl, 2020), corresponding to deterministic processes (selection) in community assembly (Xiong et al., 2017; Xiao et al., 2021). It can be inferred that stronger host filtering may lead to a more deterministic assembly, ultimately limiting the diversity of gut bacterial communities. One example of this is that a lower stomach pH can act as a filter, potentially reducing the diversity of gut microbiota (Reese and Dunn, 2018).

Thus, the core objectives of this study are to elucidate how assembly processes shape freshwater snail gut microbiota. We investigated the gut bacterial communities of two freshwater snails, the invasive snail (*Pomacea canaliculata*) and the native snail (Viviparidae), co-existing across different sites and thus exposed to the same pool of potential microbial colonizers. Although previous studies have reported on the gut bacterial communities of these two snails, their assembly patterns remain unknown, which is crucial for understanding how bacteria coexist in snail gut. To achieve this, we first comprehensively compared the differences in diversity and composition of the gut microbiota between these two snails. Subsequently, we attempted to decipher the observed differences, especially in diversity, using community assembly theory.

## 2 Materials and methods

### 2.1 Sample collection

**Figure 1:**
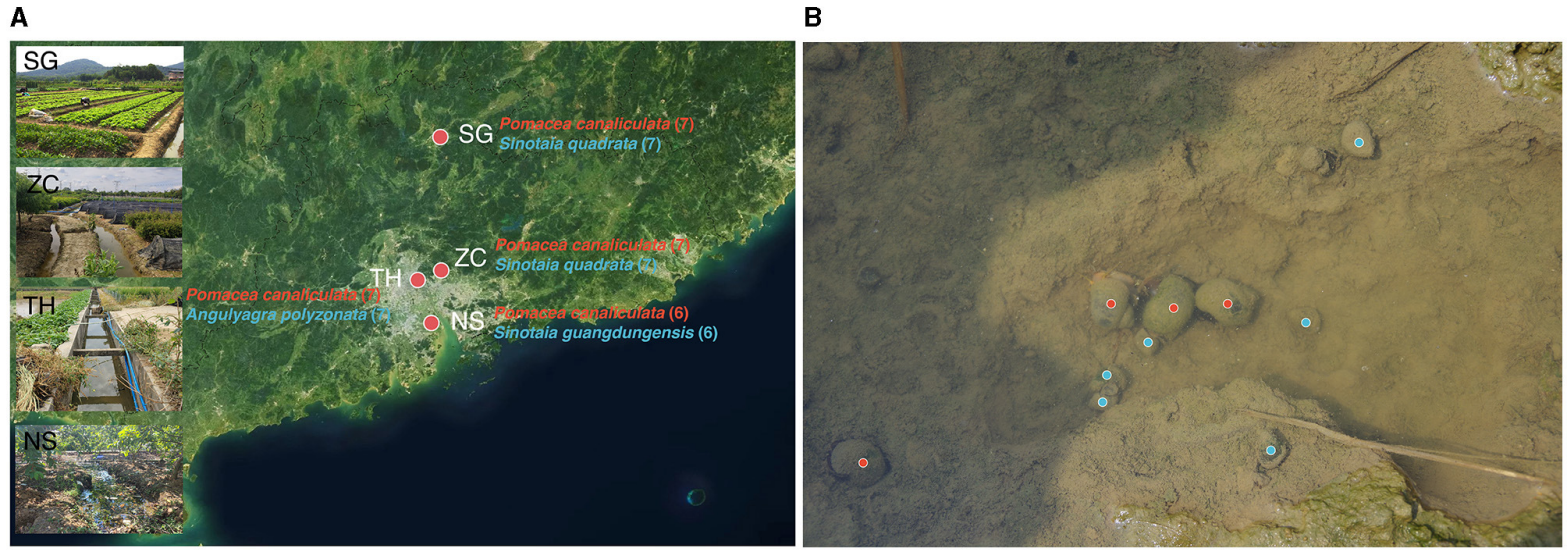
Sampling map and photo. **(A)** Sampling map displaying names of invasive and native snails for each sampling point, along with respective sample sizes in parentheses. **(B)** Photo of invasive and native snails coexisting, invasive snails and native snails were marked in red and blue, respectively.

The sample collection was conducted in Guangdong Province, China, which was one of the earliest and most severely affected areas by the invasion of golden apple snails (Shea, 1994; Yang et al., 2018; Yin et al., 2022). Four sites spanning from north to south of the province were selected, covering a distance of up to 250 km: site SG (24.78°N, 113.64°E), site ZC (23.28°N, 113.65°E), site TH (23.17°N, 113.36°E), and site NS (22.68°N, 113.53°E; Figure 1A). The selection of these sites was based on the coexistence of invasive and native snails (Figure 1B).

Invasive snail species, all identified as *Pomacea canaliculata* (Lamarck, 1822) from the family Ampullariidae (order: Architaenioglossa), and native snails including *Sinotaia quadrata* (Benson, 1842), *Sinotaia guangdungensis* (Kobelt, 1906), and *Angulyagra polyzonata* (Frauenfeld, 1862) from the family Viviparidae (order: Architaenioglossa), were collected. All these native snails are native to the sampling region (Shea, 1994; Tasi et al., 2009; Ye et al., 2020). Snails were transported to the laboratory immediately after collection and dissected 5 h later. The snail shells were wiped with 75% ethanol and removed using sterilized tools on ice. The intestines, from the pylorus to the hindgut, were gently separated and transferred into 2 mL cryogenic vials and stored at −80°C.

Samples were grouped based on the observed body size variation in the field; each invasive snail sample contained three individuals, while each native snail sample contained five individuals. Due to the rarity of male snails, especially among native species, only intestinal samples from female individuals were collected to enhance comparability. In total, 54 samples were collected, with 27 from invasive snails and 27 from native snails.

### 2.2 DNA extraction

To extract genomic DNA from gut samples, the TGuide S96 Magnetic Soil/Stool DNA Kit was utilized as per the manufacturer's instructions. The extracted DNA's quality and quantity were evaluated through electrophoresis on a 1.8% agarose gel. The NanoDrop 2000 UV-Vis spectrophotometer was employed to determine both the concentration and purity of DNA.

### 2.3 16S rRNA sequencing and bioinformatic analysis

With each DNA sample, the V3-V4 region of the bacterial 16S rRNA gene was amplified with primer pairs 338F and 806R. Both the forward and reverse 16S primers were tailed with sample-specific Illumina index sequences to allow for deep sequencing. Followed by the Polymerase chain reaction (PCR) amplification (Text S1 for details), the amplicon library was paired-end sequenced (2 × 250) on an Illumina novaseq 6000. Raw data had been submitted to the NCBI SRA database with the accession number PRJNA952540.

Bioinformatics analyses were performed with QIIME 2 2022.8 (Bolyen et al., 2019). Primers in sequence data were removed viva q2-cutadapt (Martin, 2011). The sequence data were further demultiplexed and denoised with q2-demux and q2-dada2, respectively (Callahan et al., 2016). Taxonomy was assigned to ASVs using q2-feature-classifier with a pretrained Naive Bayes classifier against the SILVA 138 reference database (Yilmaz et al., 2014; Bokulich et al., 2018; Robeson et al., 2020). Contingency-based filtering was employed to filter ASVs that show up in only one sample and non-bacterial ASVs were abandoned. The ASV table was rarefied to the fewest sequences (44,565) via q2-feature-table (Weiss et al., 2017). The phylogeny tree was constructed with fasttree via q2-phylogeny (Price et al., 2010).

### 2.4 Identification of snails

Foot muscle samples were used to isolate host DNA. The COI region of mitochondrial DNA was amplified using the universal primer pair LCO1490/HCO2198 (Folmer et al., 1994). PCR (Text S2 for details) was performed, and the resulting PCR products were sequenced on an ABI 3730xL DNA Analyzer (Applied Biosystems, CA, USA). The nucleotide sequences underwent quality control analysis using Chromas (http://technelysium.com.au/wp/chromas/) and were assembled using SeqMan (https://www.dnastar.com/). Subsequently, the sequences were aligned using the MUSCLE method (Edgar, 2004), and genetic distances were calculated using the maximum composite likelihood method (Tamura et al., 2007) in MEGA (https://megasoftware.net/). Furthermore, snail species were identified by comparing the sequences to the GenBank references using BLAST (http://blast.ncbi.nlm.nih.gov/Blast.cgi). The scientific names obtained were further verified with Global Names Verifier (http://gni.globalnames.org/).

### 2.5 Statistical analysis

In line with our objectives, we initially conducted a comprehensive comparison of the diversity and structure of the gut bacteria communities between invasive and native snails. We employed the linear mixed model with site as a random factor to examine differences in alpha diversity. Non-metric multidimensional scaling (NMDS) based on Bray–Curtis distance and permutational multivariate analysis of variance (PERMANOVA) was used to assess differences in the gut bacterial community structure between invasive and native snails. Additionally, we conducted random forest analysis across all taxonomic levels to identify differential microbes in the gut microbiota of invasive versus native snails (Liu et al., 2021a). We also utilized the linear mixed model to test differences at the genus and ASV levels. At the ASV level, we computed the phylogenetic signal using Pagel's λ with the phytools package (Pagel, 1999; Revell, 2012). Furthermore, we used SparCC to construct co-occurrence networks for the gut microbiota of invasive and native snails (Friedman and Alm, 2012). We performed 1,000 bootstrap samples to estimate pseudo *p*-values, retaining only correlations with *p*-values < 0.05 and absolute correlation values exceeding 0.6.

The phylogenetic normalized stochasticity ratio (pNST) was calculated to determine the importance of stochastic processes (Ning et al., 2019). Phylogenetic-bin-based null model analysis (iCAMP) was used to further categorize stochastic and deterministic processes into different subprocesses (Ning et al., 2020). The correlation between diversity and assembly was examined using a linear mixed-effects model.

All analyses were conducted in the R language; alpha diversity and beta diversity were calculated using the vegan package (Dixon, 2003), and linear mixed models were implemented using the lmerTest package (Kuznetsova et al., 2017). The marginal *R*^2^ (*R*${\;}_{m}^{2}$), representing the proportion of total variance explained by the fixed effects in mixed models (Nakagawa et al., 2017), was reported. Figures were generated using the ggplot2 package (Wickham, 2016).

## 3 Results

### 3.1 Sequencing information and sampling efficacy

**Figure 2:**
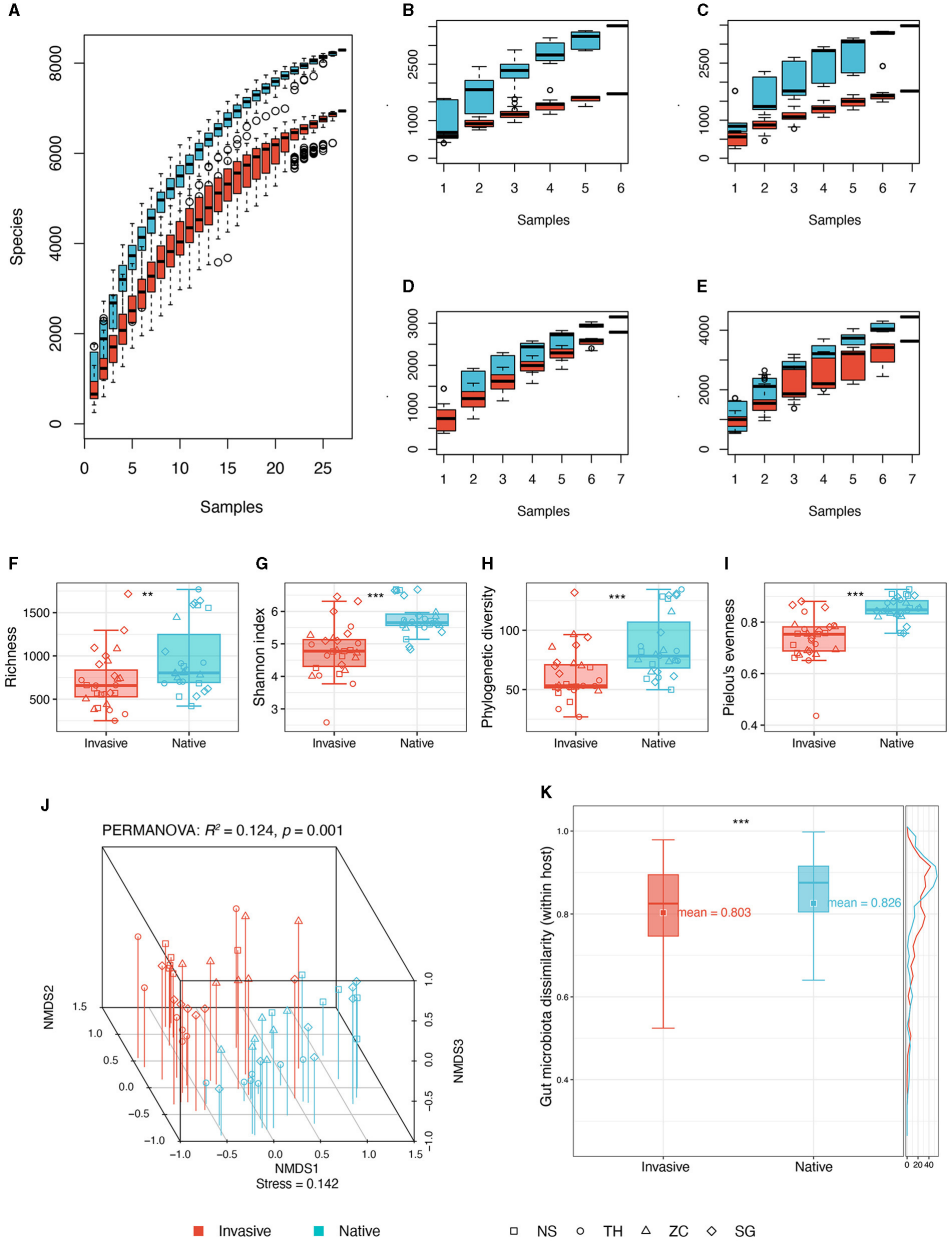
Comparisons of gut microbiota diversity between invasive and native snails. **(A)** Species accumulation curve of invasive and native snails; **(B)** Species accumulation curve of invasive and native snails in site NS; **(C)** Species accumulation curve of invasive and native snails in site TH; **(D)** Species accumulation curve of invasive and native snails in site SG; **(E)** Species accumulation curve of invasive and native snails in site ZC. **(F)** Richness; **(G)** Shannon diversity; **(H)** Phylogenetic diversity; **(I)** Pielou's evenness; **(J)** NMDS and PERMANOVA based on Bray-Curtis distance; **(K)** Dissimilarity based on Bray-Curtis distance. **p < 0.01; ***p < 0.001.

A total of 54 snail gut samples from four different sites underwent high-throughput sequencing of 16S rRNA. The analysis yielded 10,109 ASVs from 6,115,274 sequences, with per sample reads ranging from 44,565 to 182,483. Species accumulation curves suggested near saturation of ASVs for both invasive and native snails across sites (Figures 2A–E), suggesting that the sampling sufficiently captured the majority of ASVs in the snail gut within the sampling region.

### 3.2 Comprehensive comparisons of snail gut microbiota

We assessed alpha diversity using four indices: observed ASVs, Shannon, phylogenetic diversity, and evenness. All indices were significantly higher in native snails than in invasive snails (Figures 2F–I). Additionally, the gut microbiota co-occurrence network in native snails was more complex, showing a higher average degree compared with invasive snails (Supplementary Figure 1).

The NMDS plot revealed distinct differences in gut microbiota between invasive and native snails (Figure 2J). PERMANOVA confirmed these differences as significant (*R*^2^: 12.4%, *p* = 0.001). Besides, the gut microbiota of native snails displayed a minor but statistically significantly higher beta diversity compared with that of invasive snails (Figure 2K). Linear modeling found no significant correlation between genetic distances and microbiota dissimilarities among the snails (Supplementary Figure 2).

**Figure 3:**
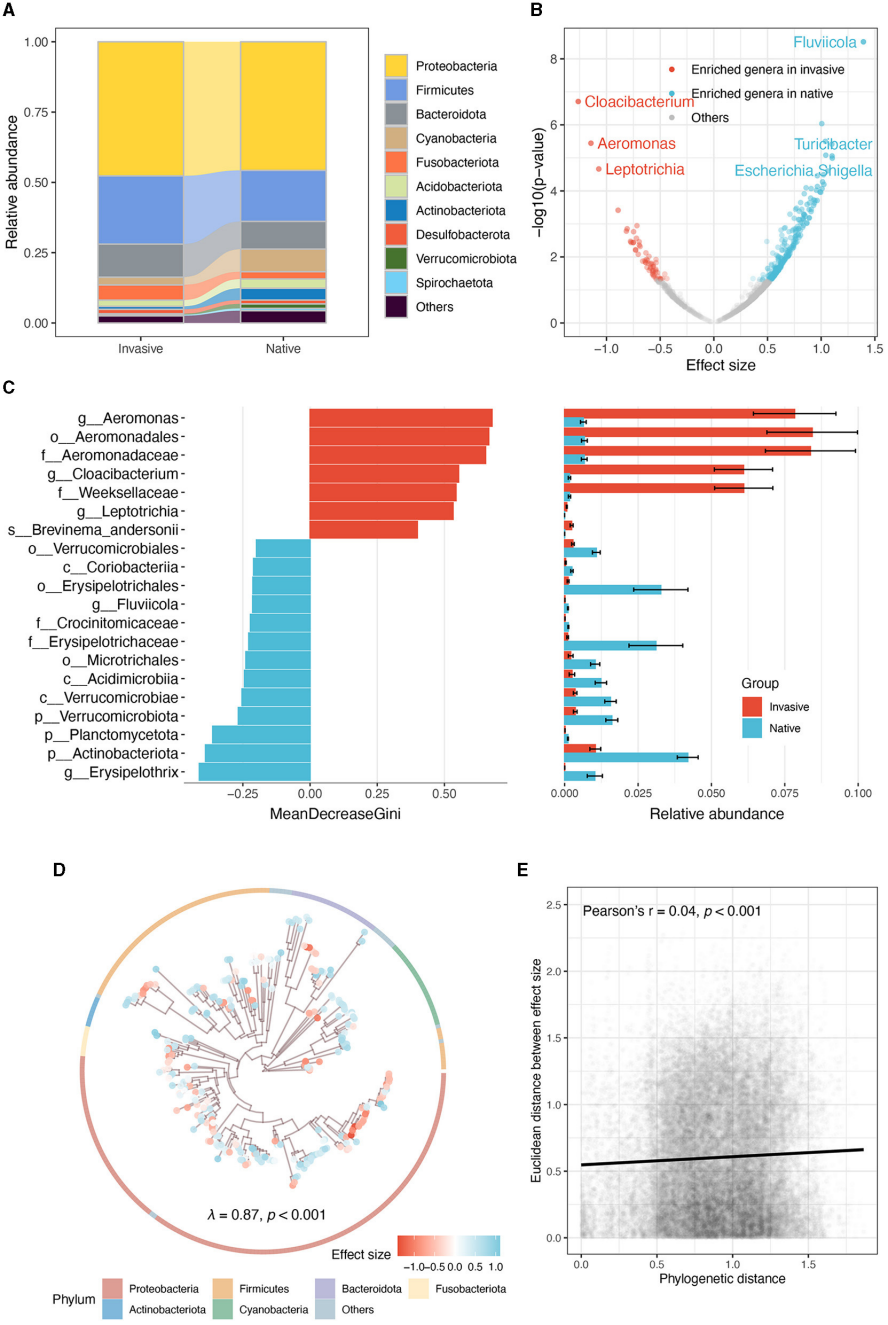
Comparative analysis of gut microbiota in invasive and native snails. **(A)** The relative abundance of dominate phyla. **(B)** Genera enriched by invasive and native snails. **(C)** Random forest analysis illustrating the top 20 microbial taxa with the largest differences. The mean and standard error of the relative abundance of these microbes in each group are displayed in the panel on the right. **(D)** Phylogenetic tree of the ASVs enriched by invasive and native snails, the tree only shows the 300 most abundant ASVs. Points at the tip are depicted to different colors according to their effect sizes (red and blue, respectively represent the enrichment of ASVs in the gut of invasive and native snails). The ring indicates the phylum of each ASV. **(E)** Correlation between phylogenetic distance and Euclidean distance among effect sizes.

At the phylum level, Proteobacteria, Firmicutes, and Bacteroidota were predominated in both snails (Figure 3A). Linear mixed model showed that native snails harbored more diverse genera (Figure 3B), including notable increases in *Fluviicola, Turicibacter*, and *Escherichia/Shigella*, while invasive snails showed significant enrichment in *Cloacibacterium, Aeromonas*, and *Leptotrichia*. Random forest analysis across all taxonomic levels further indicated significant enrichment and abundance of order Aeromonadales, family Aeromonadaceae, and family Weeksellaceae in invasive snail guts, while genus *Erysipelothrix*, phylum Actinobacteriota, and phylum Planctomycetota were much more enriched in native snails (Figure 3C).

Moreover, the gut microbes' enrichment level displayed a significant phylogenetic signal (Pagel's λ = 0.87, *p* < 0.001; Figure 3D). However, the correlation analysis showed that the phylogenetic signal was weak (Pearson's *r* = 0.04, *p* < 0.001; Figure 3E), indicating that the phylogenetic conservation is limited to several clades, rather than the entire community.

### 3.3 Assembly of snail gut microbiota

**Figure 4:**
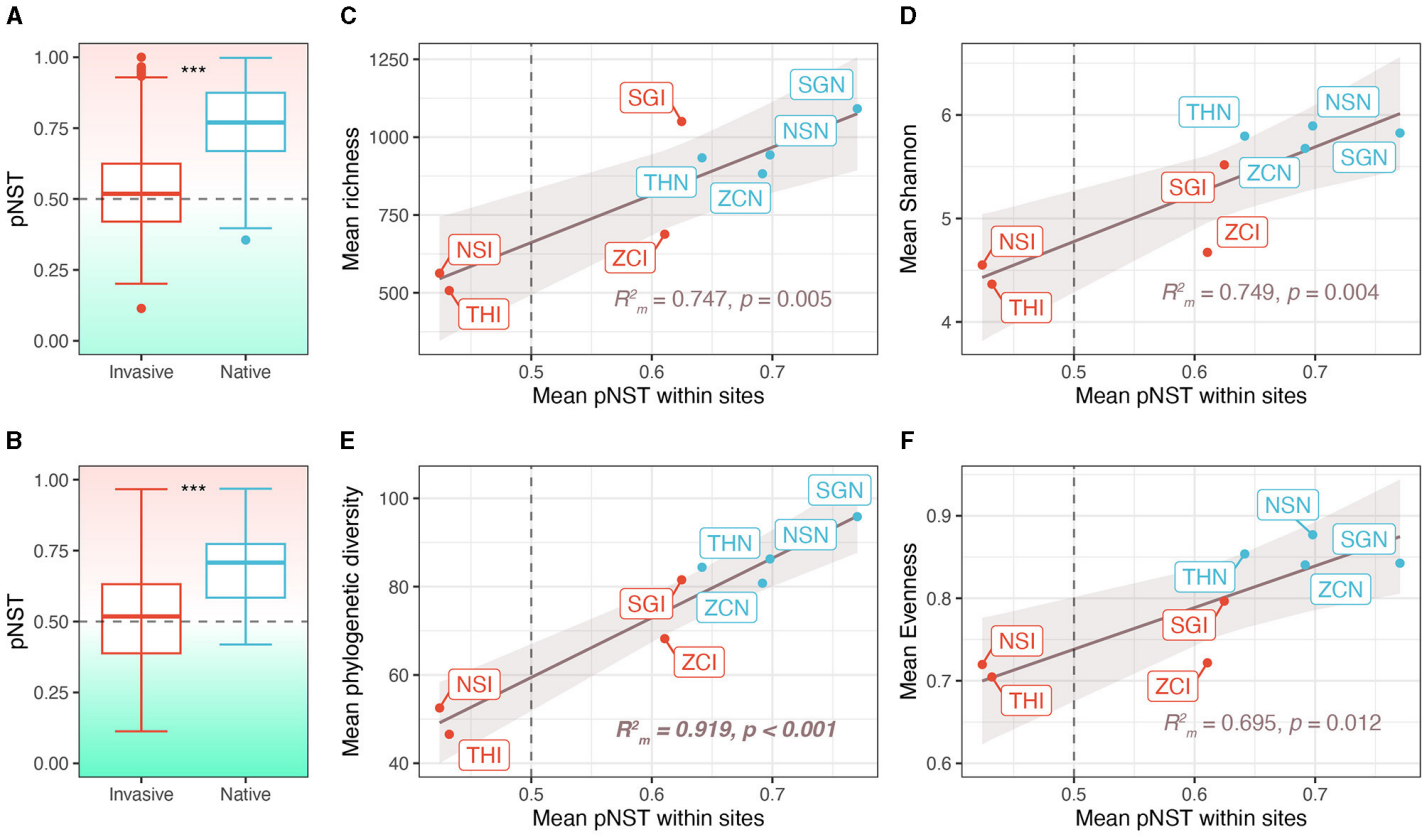
Contribution of stochastic processes to community assembly and the correlation between stochastic processes and alpha diversity. **(A)** Phylogenetic normalized stochasticity (pNST) ratio across sites. **(B)** Phylogenetic normalized stochasticity ratio within sites. **(C–F)** Correlation between pNST and alpha diversity indices. NSI, THI, SGI, and ZCI, respectively refer to the invasive snails in sites NS, TH, SG, and ZC. NSN, THN, SGN, and ZCN, respectively refer to the native snails in sites NS, TH, SG, and ZC. ***p < 0.001.

**Figure 5:**
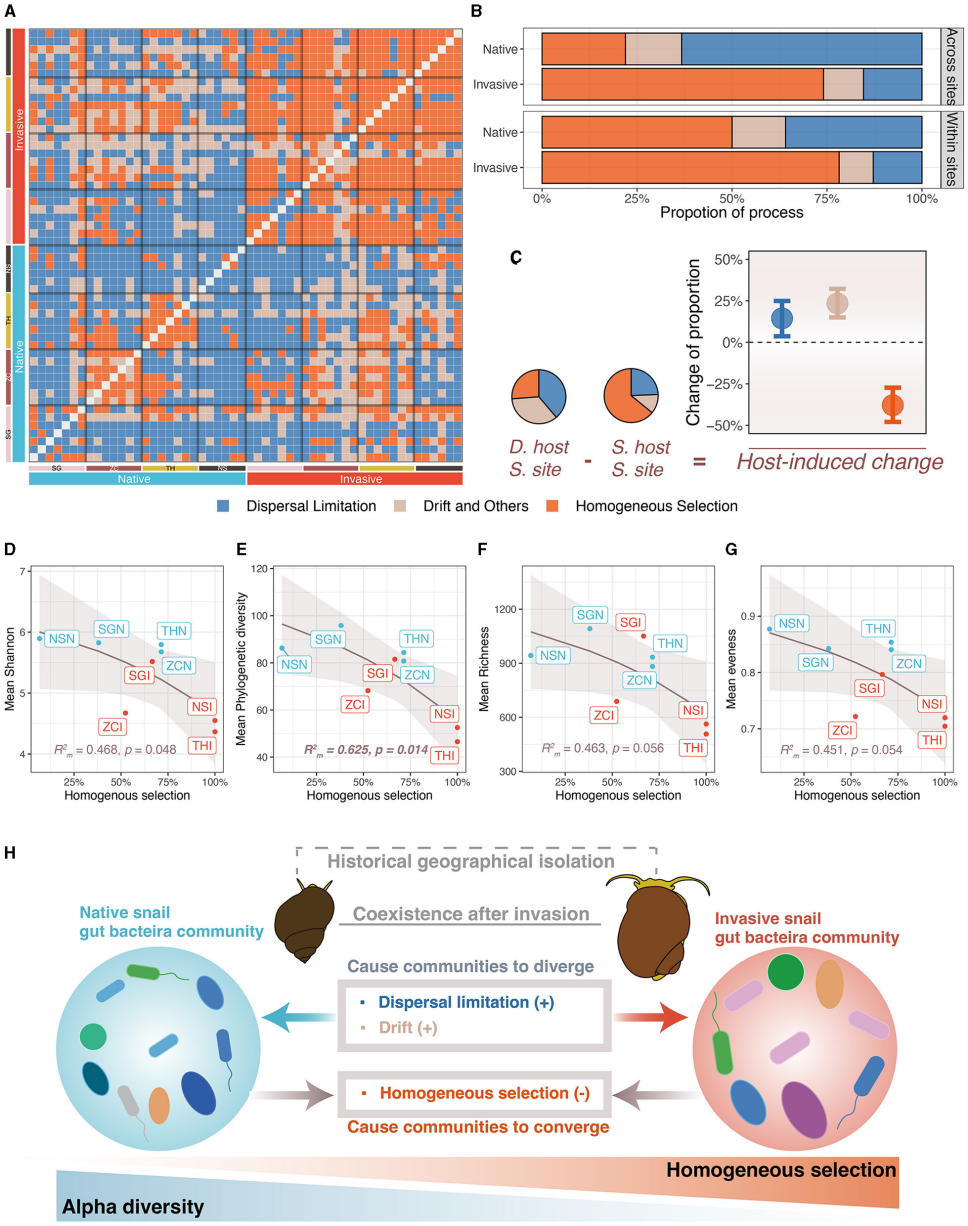
Ecological processes of freshwater snail gut microbiota assembly. **(A)** Ecological processes of paired samples. **(B)** Comparison of importance of ecological processes between invasive and native snail. **(C)** Host-induced importance changes of ecological processes (host-induced changes were calculated as different hosts at the same site minus the same host at the same site, error bars were bootstrapped 95% confidence intervals). **(D–G)** Correlation between importance of homogeneous selection and alpha diversity indexes. **(H)** Community assembly processes elucidate the differences in community structure and diversity of the freshwater gut microbiota.

The gut microbiota of native snails had significantly higher pNST compared with invasive snails both across and within sites (both *p* < 0.001, Figures 4A, B), indicating that the gut microbiota of native snails had a more stochastic assembly compared with invasive snails. The mean pNST within sites correlated significantly and positively with richness (*R*${\;}_{m}^{2}$ = 0.747, *p* = 0.005; Figure 4C), Shannon diversity (*R*${\;}_{m}^{2}$ = 0.749, *p* = 0.004; Figure 4D), phylogenetic diversity (*R*${\;}_{m}^{2}$ = 0.919, *p* < 0.001; Figure 4E), and evenness (*R*${\;}_{m}^{2}$ = 0.695, *p* = 0.012; Figure 4F). Using the iCAMP framework, we found that dispersal limitation, drift, and homogeneous selection were involved in the assembly of the snail gut microbiota (Figure 5A). Invasive snails showed a higher proportion of homogeneous selection than native snails, both within sites and across sites (Figure 5B). Besides, host-induced proportional changes in ecological processes were attributed to a significant decrease in homogeneous selection (−37.9%), as well as significant increases in dispersal limitation (14.4%) and drift (23.4%; Figure 5C). The proportion of homogeneous selection within sites was significantly and negatively correlated with Shannon diversity (*R*${\;}_{m}^{2}$ = 0.468, *p* = 0.048; Figure 5D) and phylogenetic diversity (*R*${\;}_{m}^{2}$ = 0.625, *p* = 0.014; Figure 5E), and marginally significantly negatively correlated with richness (*R*${\;}_{m}^{2}$ = 0.468, *p* = 0.056; Figure 5F) and evenness (*R*${\;}_{m}^{2}$ = 0.451, *p* = 0.054; Figure 5G).

## 4 Discussion

### 4.1 Lower diversity in invasive snail gut microbiota

The alpha diversity of gut microbiota has been extensively documented and compared across various species (Reese and Dunn, 2018; Levin et al., 2021; Zhu et al., 2022). This diversity within the gut microbiota is believed to correlate with specific host features. Studies have suggested that dietary diversity (exogenous microbial diversity) and host physiological traits (host filtering) may impact gut microbiota alpha diversity (Reese and Dunn, 2018; Santos et al., 2021). In our study, we expected the collected invasive snails, recognized as agricultural pests and attributed with a diverse diet (López-van Oosterom et al., 2016; Saveanu et al., 2017; European Food Safety Authority et al., 2020), to exhibit greater gut microbiota diversity compared with the native snails. However, our findings did not align with this expectation. This suggests that the invasive snails in our study might possess robust host filtering mechanisms, actively reducing the colonization of exogenous microbes. Additionally, our analysis of microbial networks also supports this finding. The gut microbiota network of invasive snails was simpler compared with that of native snails. Previous research on plant microbiomes has shown that host selection pressure reduces network complexity (Xiong et al., 2021), implying that invasive snails' gut microbiota may face stronger host selection pressure.

The observed lower diversity in the gut microbiota of invasive animals is not unique; several studies have reported similar findings (Minard et al., 2015; Zepeda-Paulo et al., 2018). In fact, not all gut microbes establish positive relationships with their hosts; some may act neutrally or even negatively under certain conditions (Xu et al., 2019). For instance, the gut microbe *Lactobacillus lactis* has been shown to have a negative impact on the development of *Bactrocera dorsalis* (Khaeso et al., 2018). Consequently, in studies concerning animal gut microbiota, higher diversity within the gut microbiota does not always correlate with better host fitness (Reese and Dunn, 2018).

Consistent with the results on gut microbiota diversity, we found that invasive snails enriched more bacteria compared with native snails. Both snails had Cyanobacteria in their gut microbiota, but the native snails had a higher relative abundance, suggesting that Cyanobacteria may be a food source for both types of freshwater snails, especially the native ones. At the genus level, the gut microbiota of invasive snails exhibited enrichment in *Cloacibacterium, Aeromonas*, and *Leptotrichia*. *Cloacibacterium* is found in various environments such as wastewater, sediment, and animal guts (Li et al., 2019). Notably, one species within this genus, *Cloacibacterium normanense*, has demonstrated the ability to remove heavy metals from wastewater (Nouha et al., 2016). Considering previous studies that indicate the potential accumulation of heavy metals in this snail (Hayes et al., 2015), our results suggest a plausible involvement of the invasive snail gut microbiota in heavy metal detoxification. Additionally, both *Aeromonas* and *Leptotrichia* are recognized as cellulose-degrading bacteria (Jiang et al., 2011; Liu et al., 2016). Given the significant consumption of aquatic plants by invasive snails observed in our study (European Food Safety Authority et al., 2020), the enrichment of these bacteria might indicate a focus on cellulose degradation within the gut microbiota of invasive snails. Overall, our findings suggest that the enriched gut microbiota of invasive snails may contribute to their invasiveness through processes such as heavy metal detoxification and cellulose degradation (Xu et al., 2019). However, these findings are limited by functional inference. Moving forward, acquiring direct evidence at the gene and transcript levels of gut microbiota may be crucial for further validating these findings. Additionally, in our study, the native and invasive snails belong to different families. Comparing more closely related coexisting invasive and native snails in future studies could potentially yield more profound and insightful results.

### 4.2 Deterministic assembly restricted gut microbiota diversity

The results from both pNST and iCAMP analyses showed that the assembly of the native snail gut microbiota was more stochastic compared with that of the invasive snail. This suggests that the assembly of the native snail gut microbiota is more independent of host factors (Wang et al., 2023). Subdividing the assembly process into subprocesses revealed that deterministic processes in the invasive snail mainly consisted of homogeneous selection, while stochastic processes were mainly driven by dispersal limitation. The stronger homogeneous selection in the invasive snail implies that they provide a more similar gut environment for microbial colonization (MacArthur and Wilson, 1967; Zhou and Ning, 2017), resulting in a convergence of the gut microbiota structure in invasive snails. Conversely, the higher degree of dispersal limitation in native snails indicates that the movement of gut microbes within these snails is more confined (MacArthur and Wilson, 1967; Zhou and Ning, 2017), especially between different sampling sites due to geographic isolation.

We observed a positive correlation between diversity and stochastic processes, as well as a negative correlation between diversity and homogeneous selection. While this is consistent with findings from soil microcosm experiments (Xun et al., 2019; Santillan and Wuertz, 2022), it contradicts results from a global survey of microbes across various environment types (Wang et al., 2023). Several explanations can account for the correlation between microbiota diversity and assembly processes. First, communities with lower biomass and richness are more prone to drift or founder effects (Vellend et al., 2014; Evans et al., 2017), which could amplify the significance of demographic stochastic processes. Second, higher diversity may introduce specific ecological functions that alleviate competition and offer nutrients to other microbes, thereby reducing environmental pressure and mitigating the impacts of selection (Xun et al., 2019). Third, environmental filtering may reduce microbial diversity by selectively filtering microbes, resulting in increased homogeneous selection (Xun et al., 2019). Our findings in the gut microbiota of freshwater snails are more consistent with the second and third explanations, suggesting that host selection constrains diversity across the entire gut microbiota, while communities with higher diversity alleviate the pressure of host selection due to the increase of specific ecological functions.

The observation that phylogenetic diversity accounted for the highest variation further supports the finding that host selection constrained gut microbiota diversity. A crucial aspect of host selection involves the adaptive immune system's incapacity to effectively eliminate individual gut bacteria; rather, it regulates the prevalence of microbes sharing common functional or structural traits (Ley et al., 2006). This indicates that host selection primarily filters specific microbial lineages with similar traits, potentially leading to a reduction in phylogenetic diversity.

### 4.3 Ecological processes drove the divergence of gut microbiota

Following a previous framework, we analyzed the ecological processes that differentiated host types. We found that homogeneous selection significantly decreased while dispersal limitation and drift significantly increased, which is in line with the previous suggestion that homogeneous section caused community to converge, while dispersal limitation and drift caused community to diverge (Ge et al., 2021). In our study, the greatest change was observed in homogeneous selection, implying that the lack of homogeneous selection between the two snails largely contributed to their dissimilarity. This may suggest conservative gut niches within each snail type (Wiens et al., 2010; Ge et al., 2021), especially within invasive snails (with a higher portion of homogeneous selection). Drift can trigger random birth and death events, causing stochastic fluctuations within a community and resulting in alterations to its structure (Kohl, 2020; Ge et al., 2021). However, theoretical research suggests that stochastic processes are less likely to contribute to the structure of the gut microbiota because the host's selection on microbes or competition between microbes is usually considerable, suppressing the effects of drift (Zeng et al., 2017). This implies that drift might not be the sole determinant of community structure (Kohl, 2020), which aligns with our finding indicating that drift is not the only nor the most significant factor. The limited dispersal of gut microbiota between two species reflects, on one hand, the historical-geographical isolation of their hosts. On the other hand, each snail's gut serves as an isolated small habitat. Migration and dispersal limitations between such isolated habitats are not uncommon (Sun et al., 2021). Within the gut microbiota, for microbes to migrate from one snail's gut to another, they must first survive and establish themselves in the surrounding water and sediments, and then be ingested to colonize another snail's gut; under natural field conditions, this process can be challenging.

Overall, our results demonstrated that a combination of multiple ecological processes, including both stochastic and deterministic processes, collectively drove the observed differences in the gut microbiota structure of invasive and native freshwater snails.

## 5 Conclusion

Through sampling invasive and native snails from different sites, we found that homogeneous selection, dispersal limitation, and drift govern the assembly of freshwater snail gut microbiota. Compared with native snails, homogeneous selection was more important in shaping the gut microbiota of invasive snails. This deterministic process reveals the host's filtering effect on specific microbe lineages and restricts alpha diversity, resulting in lower alpha diversity within the gut microbiota of invasive snails. Additionally, drift, dispersal limitation, and the absence of homogeneous selection resulted in differences in the structure of gut microbiota between these two snail types. Our study suggests that the assembly processes of gut microbiota are crucial in explaining variations in gut microbiota diversity and structure. Consequently, understanding these assembly processes can offer insights into microbiota-host relationships.

## Data availability statement

The datasets presented in this study can be found in online repositories. The names of the repository/repositories and accession number(s) can be found at: https://www.ncbi.nlm.nih.gov/, PRJNA952540.

## Ethics statement

The animal study was approved by Ethics Committee for Experimental Animals of South China Agricultural University. The study was conducted in accordance with the local legislation and institutional requirements.

## Author contributions

ZS: Conceptualization, Formal analysis, Investigation, Methodology, Visualization, Writing – original draft, Writing – review & editing. FY: Investigation, Writing – review & editing. QC: Investigation, Writing – review & editing. YC: Investigation, Writing – review & editing. JZ: Conceptualization, Funding acquisition, Project administration, Supervision, Writing – review & editing. JG: Investigation, Writing – review & editing. SZ: Investigation, Writing – review & editing. CZ: Methodology, Writing – review & editing.
