# Supplementary material for: More deterministic assembly constrains the diversity of gut microbiota in freshwater snails
Source: Front Microbiol. 2024 Jul 8;15:1394463. doi: 10.3389/fmicb.2024.1394463 (PMC11260827; doi:10.3389/fmicb.2024.1394463)
Supplement: Supplementary file 1 [file Data_Sheet_1.docx]

Supplementary Material

# Supplementary Figures
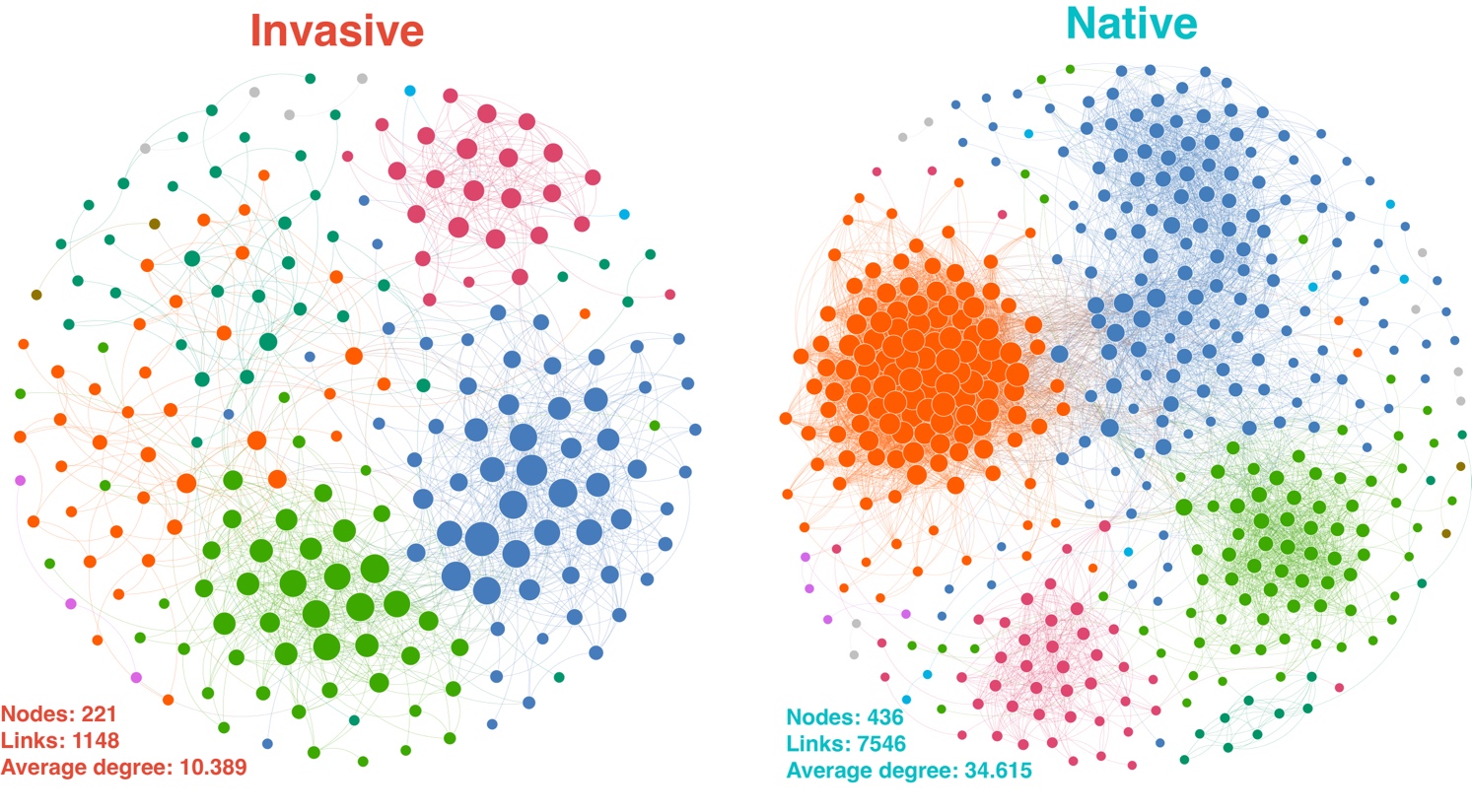


**Supplementary Figure 1.** Co-occurrence network analysis of the gut microbiota in invasive and native snails. The node colors indicate the modules to which they belong, and the node sizes correspond to the nodes' degrees.


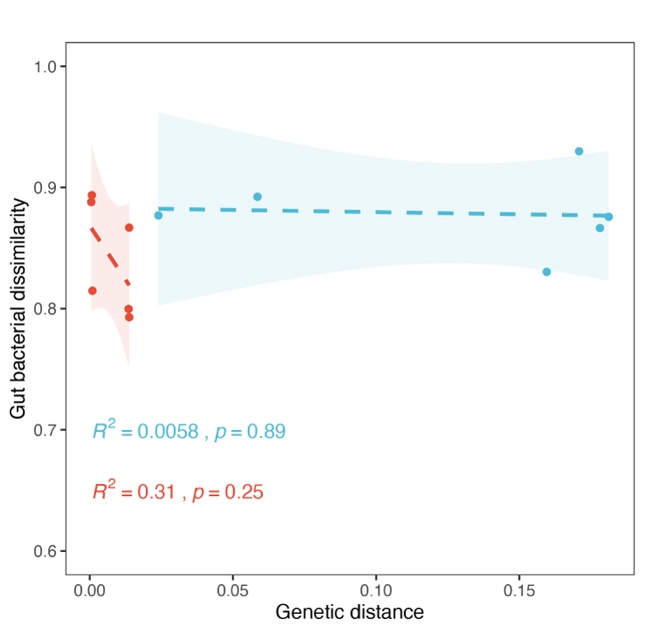


**Supplementary Figure 2.** Linear relationship between snail genetic distance and gut microbiota dissimilarity.

# Supplementary Text

**Text S1** The PCR was carried out in a total reaction volume of 10 μL: DNA template 5-50 ng, forward primer (10 μM) 0.3 μL, reverse primer (10 μM) 0.3 μL, KOD FX Neo Buffer 5 μL, dNTP (2 mM each) 2 μL, KOD FX Neo 0.2 μL, and finally ddH_2_O up to 20 μL. The PCR conditions included initial denaturation at 95°C for 5 min, followed by 20 cycles of denaturation at 95°C for 30 s, annealing at 50°C for 30 s, and extension at 72°C for 40 s, and a final step at 72°C for 7 min. After amplification, the products were purified using the Omega DNA purification kit (Omega Inc., Norcross, GA, USA) and quantified with Qsep-400 (BiOptic, Inc., New Taipei City, Taiwan, ROC).

**Text S2** The PCR was carried out in a total reaction volume of 30 μL: DNA template ~20 ng, forward primer (5 pmol/μL) 2 μL, reverse primer (5 pmol/μL) 2 μL, 2×Taq PCR Master Mix (15 μL), and finally ddH_2_O up to 10μL. The PCR conditions included initial denaturation at 95°C for 5 min, followed by 35 cycles of denaturation at 95°C for 30 s, annealing at 48°C for 30 s, and extension at 72°C for 30 s, and a final step at 72°C for 5 min.
